# Clinical heterogeneity associated with Bardet–Biedl syndrome-related genes in presumed non-syndromic inherited retinal disease

**DOI:** 10.3389/fcell.2026.1802945

**Published:** 2026-06-02

**Authors:** Bilal Azab, Dunia Aburizeg, Abdalrahman Al-Slaimieh, Rahaf Naser Aldeen, Ahmad Hyasat, Aya Alrefae, Rawand Albooz, Adnan AlAref, Ahmad Moh’d Khier Alrefae, Marya Obeidat, Azmi Hadidy, Mohammed Abu-Ameerh, Muawyah Al-Bdour, Ranad Maswadi, Ibrahim Al-Nawaiseh

**Affiliations:** 1 Department of Pathology and Microbiology and Forensic Medicine, School of Medicine, The University of Jordan, Amman, Jordan; 2 Division of Pathology and Laboratory Medicine, Phoenix Children’s Hospital, Phoenix, AZ, United States; 3 Department of Child Health, College of Medicine, University of Arizona, Phoenix, AZ, United States; 4 Ministry of Health, Amman, Jordan; 5 Department of Ophthalmology, Jordan Royal Medical Services, Amman, Jordan; 6 Jordan German Eye Center, Amman, Jordan; 7 Orthopedic Department, Jordan University Hospital, Amman, Jordan; 8 Department of Medical Laboratory Sciences, Faculty of Applied Medical Sciences, Jordan University of Science and Technology, Irbid, Jordan; 9 Department of Radiology, School of Medicine, The University of Jordan, Amman, Jordan; 10 Department of Ophthalmology, Jordan University Hospital, The University of Jordan, Amman, Jordan; 11 Department of Ophthalmology, Nottingham University Hospitals NHS Trust, Nottingham, United Kingdom; 12 Department of Surgery (Ophthalmology), King Hussein Cancer Centre (KHCC), Amman, Jordan

**Keywords:** Bardet–Biedl syndrome, ciliopathies, genetic testing, inherited retinal disease, reverse phenotyping, Beales, InterEuropean Reference Networks

## Abstract

**Introduction:**

Inherited retinal diseases (IRDs) may present as an isolated ocular condition or as part of multisystem disorders, such as Bardet–Biedl syndrome (BBS). Several BBS-related manifestations are age-dependent and variably expressed. Therefore, patients with early or subtle extra-ocular features may be incorrectly diagnosed as having non-syndromic IRD. There are two diagnostic frameworks for BBS: a phenotype-based, Beales-derived approach and a genotype-first approach, as recommended by the InterEuropean Reference Networks (ERNs) criteria. Here, we aimed to conduct comprehensive evaluations of patients with IRD carrying BBS-related variants.

**Methods:**

Nineteen patients from nine Jordanian families initially diagnosed with non-syndromic IRD underwent detailed ophthalmic evaluation and molecular testing. Post-genetic testing, participants underwent targeted reverse phenotyping to reveal potentially overlooked syndromic features.

**Results:**

Ophthalmic assessment revealed a spectrum ranging from advanced IRDs to phenotypes inclining toward rod–cone, cone–rod, and macular dystrophies. Molecular testing identified potential causative variants in BBS-related genes known to cause both isolated IRD and BBS, namely, *CFAP418*, *BBS2*, *BBS1*, *BBS5*, and *CEP290*. A splice donor variant in *CFAP418* was the most frequent in our cohort (numbers [n] = 3/9 families), followed by a missense variant in *BBS2* (n = 2/9 families). These findings prompted genotype-guided reverse phenotyping, which uncovered previously unrecognized multisystemic involvement. BBS-related major and minor features were discovered across the cohort. The extraretinal major features included obesity (n = 16 patients), polydactyly (n = 7 patients), renal anomalies (n = 7 patients), genitourinary abnormalities (n = 4 patients), and intellectual disability (n = 4 patients). Using Beales-derived criteria, 11 patients met the threshold for BBS. Under ERNs genotype-first recommendations, 18 patients reached the BBS diagnostic limit.

**Discussion:**

Wide intra- and inter-familial clinical variability was observed, even among patients with the same variants in *CFAP418* and *BBS2*. Furthermore, applying genotype-guided diagnostic criteria reclassified multiple patients who did not meet the traditional Beales-based clinical criteria as having BBS. These findings demonstrate that a genotype-first approach can detect patients with unrecognized BBS and facilitate appropriate clinical surveillance, genetic counseling, and management. To our knowledge, this is the first study from Jordan to integrate molecular testing with reverse phenotyping in patients carrying BBS-related genotypes. Importantly, we present the most comprehensive extra-retinal characterization of patients with *CFAP418*, so far.

## 1 Introduction

Inherited retinal diseases (IRDs) are degenerative disorders characterized by the loss of photoreceptors or the retinal pigment epithelium (RPE) (Ben-Yosef, 2022). Clinically, IRDs may present as isolated retinal conditions or as features of multisystem diseases (Ben-Yosef, 2022). Notably, more than 25% of all IRDs emerge from defects in cilia-related proteins, with Bardet–Biedl syndrome (BBS) representing a prototypical retinal ciliopathy (Delvallée and Dollfus, 2023). BBS is an autosomal recessive disorder with approximately 26 genes implicated so far (Tomlinson, 2024). BBS has an estimated prevalence ranging between 1:160,000 in Northern European populations and 1:13,500 in Bedouins (Priya et al., 2016). The BBS diagnosis follows two frameworks: the traditional, phenotype-based approach adapted from Beales et al. and the more recent genotype-first model recommended by the InterEuropean Reference Networks (ERNs) (Forsyth and Gunay-Aygun, 2023; Melluso et al., 2023; Dollfus et al., 2024). The classical phenotype-based classification, influenced by Beales et al., requires at least four major features or a combination of a minimum of three major and two minor features (Beales et al., 1999; Forsyth and Gunay-Aygun, 2023; Melluso et al., 2023). The major BBS-related features include IRD, obesity, post-axial polydactyly (PAP), renal anomalies, genitourinary abnormalities, and learning disability (Forsyth and Gunay-Aygun, 2023; Melluso et al., 2023). The BBS-related minor features are more heterogeneous and less characterized, including brachydactyly, syndactyly, distinctive craniofacial features, endocrine and metabolic abnormalities, and behavioral, psychiatric, and associated neurologic features (Forsyth and Gunay-Aygun, 2023; Melluso et al., 2023).

Given the progressive nature of several BBS-related features, the traditional framework falls short in enabling early recognition and timely diagnosis (Dollfus et al., 2024). For instance, IRDs, weight gain, and kidney diseases may initially be subtle or subclinical, leading to underdiagnosis (Dollfus et al., 2024; Forsyth and Gunay-Aygun, 2023). Moreover, BBS-associated manifestations exhibit variable expressivity and marked intra- and inter-familial variability, limiting the ability to reach the traditional diagnostic thresholds for BBS (Nawaz et al., 2023). Interestingly, IRDs are attributed as the most penetrant feature of BBS; hence, patients with overlooked extra-ocular features could be mistaken as a case of isolated IRD, rather than syndromic (Dollfus et al., 2024; Nawaz et al., 2023). Therefore, using a genotype-based framework for BBS diagnosis may reveal masquerading BBS-associated features, reduce diagnostic delay, and facilitate earlier management (Dollfus et al., 2024). ERNs have recommended a diagnostic scheme for BBS that incorporates genetic findings into the diagnostic process (Dollfus et al., 2024). Under the ERNs’ recommendations, a diagnosis of BBS can be established owing to the presence of disease-causing variants in BBS-related genes, along with a selected set of BBS-related clinical features (Dollfus et al., 2024). This genotype-first approach reflects less stringent diagnostic criteria to establish BBS diagnosis compared with traditional phenotype-based frameworks (Dollfus et al., 2024).

In Arab countries, the BBS burden has been estimated to account for 30% of syndromic IRDs (Jaffal et al., 2023). So far, the prevalence of BBS in Arabs has not been fully documented. An estimated BBS prevalence of approximately 1 in 43,474 can be inferred from a hospital-based large-scale study in Egypt (Tawfik et al., 2023). Furthermore, BBS accounted for 28.2% of all syndromic IRD cases in Egypt, closely mirroring the broader regional estimate (Tawfik et al., 2023). BBS remains underreported in Jordan, with only a single case described so far that lacked genetic testing (Hassona et al., 2017). Notably, *CFAP418* has been rarely implicated in BBS, with limited reported cases and incomplete characterization of its extraretinal manifestations; accordingly, further clinical delineation of *CFAP418*-associated disease has been recommended by ERNs.

In our work, we aimed to (1) characterize intra- and inter-familial clinical variability in patients with presumed non-syndromic IRD harboring variants in BBS-related genes; (2) conduct a genotype-guided reverse phenotyping approach to detect potential masquerading syndromic features; and (3) assess patient classification for BBS under both traditional phenotype-based and ERNs-recommended genotype-first diagnostic frameworks. To our knowledge, our work is the first to use molecular testing and reverse phenotyping assessment in patients with BBS-related genes in Jordan. Moreover, given the rarity of BBS cases with *CFAP418*, our study findings provide additional extra-ocular characterization of patients with *CFAP418*, contributing to expanding the clinical spectrum associated with this gene.

## 2 Methods

### 2.1 Study design and ophthalmic evaluation

This is a retrospective, genotype-driven observational study derived from a larger cohort of 100 families with IRD, a subset of which was reported in our earlier work (Azab et al., 2021). IRD diagnoses were based on documented medical records, together with detailed ophthalmic history and clinical features consistent with the disorder, such as progressive visual impairment, nyctalopia, hemeralopia, photophobia, color vision abnormalities, or peripheral or central vision loss. As baseline evaluations were strictly ophthalmic, patients were initially presumed to have isolated IRD.

**FIGURE 1:**
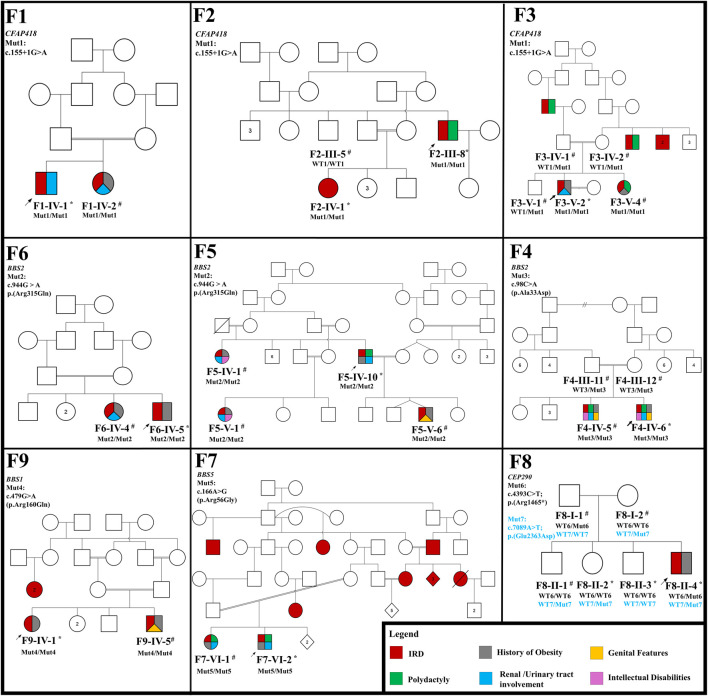
Pedigrees for families from F1 to F9 carrying variants in Bardet-Biedl syndrome-related genes. Squares, circles, diamonds, and double horizontal lines represent male individuals, female individuals, individuals of unknown gender, and consanguinity, respectively. Certain clinical features are represented by filled sectors within symbols, as represented according to the color key: red denotes inherited retinal dystrophy (IRD); green denotes polydactyly; gray denotes a history of obesity; blue denotes renal/urinary tract involvement, including major renal anomalies, namely renal cysts and hydronephrosis, along with additional urinary tract features such as neurogenic bladder (a minor feature), chronic cystitis, and extrarenal pelvis; yellow denotes genital features; and pink denotes intellectual disability. The genotype of each tested individual is shown below the corresponding ID (Mut, mutant; WT, wild-type). An arrow indicates the proband. Individuals primarily tested through exome sequencing and Sanger sequencing are marked by an asterisk (*) and hash symbol (#), respectively, shown as superscripts at the upper right of the IDs. The identified variants are as follows: Mut1: *CFAP418:*c.155 + 1G >A (F1, F2, F3); Mut2: *BBS2:*c.944G >A, p.Arg315Gln (F5, F6); Mut3: *BBS2:*c.98C >A, p.Ala33Asp (F4); Mut4: *BBS1:*c.479G >A, p.Arg160Gln (F9); Mut5: *BBS5:*c.166A >G, p.Arg56Gly (F7); Mut6: *CEP290:*c.4393C >T, p.Arg1465*; and Mut7: *CEP290:*c.7089A >T, p.Glu2363Asp (F8).

Subsequent molecular testing of the larger cohort identified 19 patients from nine families (F1–F9) harboring variants in BBS-related genes (Figure 1). These families were retrospectively included in the present investigation to evaluate potential syndromic involvement by conducting genotype-guided reverse-phenotyping assessments. Inclusion in this sub-cohort required a documented baseline diagnosis of IRD, alongside molecular confirmation of BBS-related variants. Since the baseline evaluations were strictly ophthalmic, the sole exclusion criterion was the absence of BBS-related variants upon molecular testing.

For 16 out of 19 patients, IRD diagnosis was further supported by in-person ophthalmic assessment conducted by our team (RN, MA-A, MA-B, RM, and IA-N) at Jordan University Hospital and/or the Jordan German Eye Center. Ophthalmic assessment included evaluation of unaided visual acuity (VA) and best-corrected visual acuity (BCVA) using an E-illiterate optotype, recorded in decimal notation, along with objective and subjective refraction. Color vision testing was performed using the Ishihara color vision test (14-plate edition). After pupillary dilation with 1% tropicamide, true-color fundus photography, fundus autofluorescence (FAF), and optical coherence tomography (OCT) foveal 7 × 7 mm scanning protocols, were performed using the Topcon Triton Swept-Source OCT (Topcon Corporation, Tokyo, Japan). Slit-lamp biomicroscopy assessment was performed when feasible. Of note, the assessment of three patients (F3-V-4, F9-IV-1, and F9-IV-5) was done retrospectively by reviewing their provided medical records and history as they declined to undergo further ophthalmic assessment.

Four patients underwent electroretinography (ERG), including pattern ERG (PERG) and full-field ERG (ffERG), in accordance with the standards of the International Society for Clinical Electrophysiology of Vision (ISCEV) (Robson et al., 2022; Thompson et al., 2024). Recordings were obtained using the RETI-port/scan 21 system (Roland Consult, Brandenburg, Germany). A Dawson, Trick, and Litzkow (DTL) fiber electrode, a sterile ERG thread electrode, was used as an active electrode, with reference electroencephalography (EEG) gold electrodes placed at the outer canthus and a ground EEG gold electrode positioned on the forehead.

This study was approved by the Institutional Review Board (IRB) of the Jordan University Hospital (Protocol code 2018/84, dated 2018/2/20) and Deanship of Scientific Research (Decision No. 433/2025, dated 2025/05/12) and in concordance with the recommendations of the Declaration of Helsinki. Written informed consent was obtained from all participants before inclusion.

### 2.2 Genetic testing

Peripheral blood samples were collected using ethylenediaminetetraacetic acid (EDTA) tubes from the affected individuals and their available relatives. DNA extraction was conducted using the Promega Wizard DNA extraction kit following the provider’s instructions (Madison, Wisconsin, United States). The probands’ samples from each family were subjected to whole-exome sequencing (WES) using the Agilent SureSelect Human All Exon 65 Mb kit V5 (Santa Clara, California, United States), Agilent SureSelect V6 post, or TwistBioscience, as previously described (Azab et al., 2021). The constructed libraries were sequenced using either Illumina HiSeq 2500 or NovaSeq 6000 platforms. A multi-tiered filtration process for variant prioritization was followed as previously outlined (Azab et al., 2021). Several family members underwent co-segregation analysis through Sanger sequencing or WES (Figure 1; Supplementary Figure S1).

### 2.3 Reverse clinical assessment

Following the discovery of BBS-related variants through genetic testing, a physician (AA-S, general practitioner) conducted a detailed reverse-phenotyping. This comprehensive physical and historical assessment aimed to identify both major and minor BBS criteria across multiple clinical domains. Specifically, the evaluation encompassed the following: (1) anthropometric measurements and weight trajectory history, including body mass index (BMI) calculations and assessment for early-onset obesity and hyperphagia; (2) a craniofacial assessment, comprising head circumference (HC) measurements and structural evaluation for dysmorphic features affecting the skull, face, neck, and oral cavity; (3) an extremity and musculoskeletal examination to identify major anomalies such as PAP, as well as minor limb features and gait abnormalities; (4) a genitourinary and pubertal assessment evaluating features such as genital hypoplasia, cryptorchidism, and history of urologic complications; (5) neurodevelopmental and behavioral screening to assess for global developmental delay, intellectual disability, speech/motor delays, and psychiatric manifestations; and (6) a review of endocrine and metabolic abnormalities, including insulin resistance (INR) and dyslipidemia, when available. Notably, intellectual disability was recorded based on clinical assessment, caregiver input, and retrospective chart review and was not stratified into different categories of severity due to the retrospective nature of the data and lack of standardized psychometric evaluation.

To complement the physical examination, post-genetic testing, targeted imaging was used to reveal structural features in the hands (H-), feet (F-), and urinary tract (UT), using H-F-X-ray (XR) and UT- ultrasound (UT-US), respectively. Scrotal US was evaluated if recommended by the clinician for selected patients. Three individuals, F3-V-4, F9-IV-1, and F9-IV-5, declined to undergo formal clinical evaluation, including craniofacial assessment. The available information for these individuals was self-declared (F9-IV-1 and F9-IV-5), obtained from medical records (F3-V-4), or both (F3-V-4).

### 2.4 Diagnostic classification of BBS

Each recruited individual underwent BBS diagnosis by use of two diagnostic frameworks. The first was the traditional, phenotype-based approach derived from Beales et al. (1999), as summarized in GeneReviews and by Melluso et al. (2023) and Forsyth and Gunay-Aygun (2023). The second was the genotype-guided 2024 consensus recommendations issued by ERNs (Dollfus et al., 2024). Classification under both frameworks was applied retrospectively using the available phenotypic and molecular data.

## 3 Results

### 3.1 Overview

We included 9 families comprising 19 affected individuals (11 male and 8 female individuals). All patients were previously presumed to have isolated IRD without clinical suspicion of syndromic involvement (Figure 1). In this study, we conducted a thorough ophthalmic assessment followed by genetic testing. Variants were identified in five genes associated with either isolated IRD or BBS (Table 1). In short, three families (F1–F3) carried the same causative variant in *CFAP418.* Three other families (F4–F6) harbored variants in *BBS2*, two of which (F5 and F6) shared the same variant. The remaining families had variants in *BBS5* (F7), *CEP290* (F8), and *BBS1* (F9).

**TABLE 1:** Details of the identified variants in Bardet–Biedl syndrome-related genes.

Family ID	Gene	Variant coordinates (hg38 and hg19)	HGVS nomenclature	Zygosity	MAF in the gnomAD Population database V4 and V2	Co-seg.?	*In silico* prediction REVEL (RL) and AlphaMissense (AM)	ClinVar ID classification	ACMG classification (criteria)
F1–F3	*CFAP418*	hg38:chr8:95269034 hg19:chr8:96281262	NM_177965.3:c.155 + 1G>A	HOM	v2:NA v4:0.00001667 (Admixed American)	Yes	SpliceAI 0.95	Not listed	Pathogenic (PVS1, PM2_P, PP1_M, and PP4)
F4	*BBS2*	hg38:chr16:56519765 hg19:chr16:56553677	NM_031885.3:c.98C>A (p.Ala33Asp)	HOM	v2:0.00002907 (Admixed American) v4:0.00001668 (Admixed American)	Yes	RL: 0.872 AM:0.99	209042 (LP; P; VUS)	Pathogenic (PM2_P, PP1_S, PM3_S, PP4, and PP3)
F5-F6	*BBS2*	hg38:chr16:56502453 hg19:chr16:56536365	NM_031885.3:c.944G>A (p.Arg315Gln)	HOM	v2:NA v4:0.00002196 (South Asian)	Yes	RL:0.862 AM:0.332	555022 (P; LP)	Pathogenic (PM3_VS, PM5, PM2_P, PS3_M, PP1, PP3, and PP4)
F7	*BBS5*	hg38:chr2:169487092 hg19:chr2:170343602	NM_152384.3:c.166A>G (p.Arg56Gly)	HOM	v2:NA v4:NA	Yes	RL:0.92 AM:0.992	585188 (P; LP)	Pathogenic (PM3_S, PS3_M, PM2_P, PP3, and PP4)
F8	*CEP290*	hg38:chr12:88086083 hg19:chr12:88479860	NM_025114.3:c.4393C>T (p.Arg1465*)	ComHET	v2:0.00005678 (Admixed American) v4:0.00003338 (Admixed American)	Yes	-	217626 (P)	Pathogenic (PVS1, PM2_P, PM3_VS, PP1_S, and PP4)
		hg38:chr12:88053692 hg19:chr12:88447469	NM_025114.3:c.7089A>T (p.Glu2363Asp)	ComHET	v2:NA v4:0.000006972 [European (non-Finnish)]	Yes	RL:0.099 AM:0.141	Not listed	VUS (PM2_P, BP4, and PP4)
F9	*BBS1*	hg38:chr11:66515586 hg19:chr11:66283057	NM_024649.5:c.479G>A (p.Arg160Gln)	HOM	v2:0.0003076 (African/African American) v4:0.0001644 (Middle Eastern)	Yes	RL:0.599 AM:0.106	370228 (P; LP)	Pathogenic (PM2_P, PM3, PP1_M, PS3, and PP4)

Abbreviations: ACMG, American College of Medical Genetics and Genomics; AM, AlphaMissense score; Co-seg.; co-segregation analysis; ComHET, compound heterozygous; gnomAD, Genome Aggregation database; hg19, human genome assembly GRCh37; hg38, human genome assembly GRCh38; HGVS, Human Genome Variation Society; HOM, homozygous; LP, likely pathogenic; MAF, minor allele frequency; NA, not available; P, pathogenic; RL, REVEL score; v2, gnomAD version 2.1.1; v4, gnomAD version 4.1.1; VUS, variant of uncertain significance. Amino acids: Ala, alanine; Asp, aspartic acid; Glu, glutamic acid; Gln, glutamine; Arg, arginine; *, indicates a stop codon.

Following the genetic investigation, to assess the plausible syndromic involvement of these genes, we conducted a genotype-guided reverse phenotyping, which included comprehensive systemic examination and imaging through H–F-XR and UT-US, aiming to identify overt or subtle syndromic features that were not identified during initial evaluations. Overall, the reverse phenotyping assessment revealed a wide spectrum of extra-retinal manifestations across the cohort (Table 2). The most prevalent major BBS-related features identified were obesity (n = 16), followed by PAP (n = 7), renal anomalies, including hydronephrosis, and cysts, represent major renal features (n = 7), genitourinary abnormalities (n = 4), and intellectual disability (n = 4).

**TABLE 2:** Comprehensive ophthalmic, systemic, and diagnostic characterization of patients with BBS-related variants.

Patient ID (Age^1^ and sex) Gene: variant	IRD	Anthropometrics and obesity Hx (W (kg)/H (cm)) BMI	Polydactyly and other MSK	CF (HC)	GU Puberty ENDO	NB Comorbidities and additional features	H–F-XR	UT-US	Major minor BBS criteria Beales/ERNs
F1-IV-1 (17 M) *CFAP418*: c.155 + 1G>A	Likely RCD	W:74/H:176 BMI:23.9 NAp; no early-onset weight gain	None	High anterior hairline, epicanthal fold, and broad nasal bridge (HC 58 cm)	Normal	Normal NB Hx of prenatal RC	H-XR: Normal F-XR: BL short MP of the T5; Lt os naviculares	Rt moderate HN	Major: RD and REN Minor: CF and MSK Beales: No ERNs: Yes
F1-IV-2 (15 F) *CFAP418*: c.155 + 1G>A	Likely RCD	W:72/H:156 BMI: 29.6 Progressive weight, and gain in adolescence (>95th percentile for age)	None	Epicanthal fold (HC 54 cm)	Normal GU and puberty INR, dyslipidemia, and PCOS	None	H-XR: Normal F-XR: BL two Ph of the T5; BL T5 Clino.	Normal (Lt ERP)	Major: RD and OB Minor: CF, ENDO, and MSK Beales: No ERNs: Yes
F2-III-8 (36 M) *CFAP418*: c.155 + 1G>A	Advanced; likely CRD	W:70/H:173 BMI: 23.4 Normal weight trajectory, NAp	LL PAP None	Unilateral epicanthal fold (HC 54 cm)	Normal	None	H-XR: Normal F-XR: PAP of the Rt T5. (sixth toe consisting of two Ph) BL Clino of the T5	Normal	Major: RD and PAP Minor: CF and MSK Beales: No ERNs: Yes
F2-IV-1 (11 F) *CFAP418*: c.155 + 1G>A	CRD	W:33/H:140 BMI:16.8 Stable, NAp	None BL pes planus	Micrognathia (HC 51 cm)	Normal Tanner 2	None	H-XR: Normal F-XR: BL two Ph of the T5; triangular shape of the distal phalanx of the T2, T3, and T4 (BL)	Normal	Major: RD Minor: CF and MSK Beales: No ERNs: Yes
F3-V-2 (44 M) *CFAP418*: c.155 + 1G>A	Advanced; likely CRD	W:134/H:195 BMI:35.2 Progressive weight gain into adolescence; NAp	None Tall stature; tall wingspan, truncal obesity, and proximal muscle wasting	Occipital flattening, high anterior hairline, thin upper lip, and prominent chin (HC 58 cm)	Normal GU and puberty Dyslipidemia	Depression (on escitalopram) OSA and chronic cough/wheeze	H-XR: suspected Boutonniere deformities F-XR: BL two Ph of the T5	BL RS (1.3 cm + 1.1 cm) Lt RC (1.6 × 1.1 cm)	Major: RD, OB, and REN Minor: CF, ENDO, NB, and MSK Beales: Yes ERNs: Yes
F3-V-4 (50 F) *CFAP418*: c.155 + 1G>A	Reported IRD, NA	W:117/H:165 BMI:43 early-childhood obesity	BL UL&LL PAP (surgically removed) Gait abnormality (self-declared)	NA	Unspecified renal involvement (self-declared) T2DM, hypothyroidism; dyslipidemia	Social withdrawal; depression; on sertaline and aripiprazole Abnormal movements (chorea and tics); seizures, refusal of exam	NA	NA	Major: RD, OB, and PAP Minor: ENDO, and NB Beales: Yes ERNs: Yes
F4-IV-5 (32 M) *BBS2*: p.Ala33Asp	Significant maculopathy; suggestive CRD	W:87/H:173 BMI:29.1 Early-onset obesity; progressive; HP	LL PAP (surgically removed) Pes cavatum, and gait abnormality	Occipital flattening and broad nasal bridge (HC 52 cm)	Genital hypoplasia; VUR; Hx of cryptorchidism, Hypothyroidism	GDD; ID; autistic spectrum behaviors; social withdrawal and isolation HTN; seizure	H-XR: Rt F5 Clino; Short and small MP of the Rt F5 F-XR: Rt Clino on the T5; Lt residual ossicle at the T5 resembling the remnant of PAP.	Normal (BL ERP)	Major: RD, OB, PAP, ID, and GU Minor: CF, NB, ENDO, and MSK Beales: Yes ERNs: Yes
F4-IV-6 (29 M) *BBS2*: p.Ala33Asp	Significant maculopathy; suggestive CRD	W:106/H:180 BMI:32.7 Early-onset obesity; progressive; HP	UL PAP (surgically removed) Pes cavatum, and gait abnormality	Short neck (HC 55 cm)	Genital hypoplasia; VUR	GDD; ID; social anxiety, isolation, and stereotypic behaviors; autistic spectrum behaviors; asthma and snoring; unspecified kidney disease	H-XR: Normal F-XR: BL T5 Clino.	Lt moderate HN	Major: RD, OB, PAP, ID, REN, and GU Minor: CF, NB, and MSK Beales: Yes ERNs: Yes
F5-IV-1 (53 F) *BBS2*: p.Arg315Gln	Advanced; likely CRD	W:96/H:146 BMI:45 obesity since early childhood; rapid; HP	None	High anterior hairline; short neck; macroglossia (HC 61 cm)	Normal GU and puberty INR	GDD; ID; speech delay; impaired social skills; autism spectrum behaviors; impaired social skills; nearly mute Snoring	H-XR: Short Rt fourth metacarpal bone (brachymetacarpia) F-XR: Lt os peroneum; Rt os naviculare	Chronic cystitis	Major: RD, OB, and ID Minor: CF, ENDO, NB, and MSK Beales: Yes ERNs: Yes
F5-IV-10 (39 M) *BBS2*: p.Arg315Gln	Advanced; likely CRD	W:114/H:180 BMI:35 Progressive weight gain since childhood; HP	Lt UL PAP BL pes planus	Frontal bossing and deep set eyes (HC 58 cm)	Rt varicocele Suspected INR (acanthosis nigricans)	Normal NB snoring; keloid and slurred speech	H-XR: PAP of the Lt F5 (consisting of two Ph) F-XR: Two Ph of the Lt T5; BL T5 Clino; short MP of the Rt T5	Rt RC (2.3 × 1.8 cm) Few Rt renal angiomyolipoma, the largest 1.4 × 1.5 cm Dromedary hump (normal variant of the Lt kidney); chronic cystitis	Major: RD, OB, PAP, and REN Minor: CF, ENDO, and MSK Beales: Yes ERNs: Yes
F5-V-1 (15 F) *BBS2*: p.Arg315Gln	Maculopathy	W:71/H:157 BMI:28.8 (>95th percentile for age); overweight since childhood; progressive adolescent weight gain; HP	None BL pes planus; gait abnormality; suspected leg-length discrepancy	Hypertelorism; retrognathia (HC NA)	Recurrent UTIs; VUR; neurogenic bladder INR	ID; impaired social skills; Birth weight 4 kg; multiple joint pains and stiffness; undergoing rheumatologic workup	H-XR: Boutonniere deformities F-XR: Claw toe deformities	Neurogenic bladder	Major: RD, OB, and ID Minor: CF, MSK, REN, and ENDO Beales: Yes ERNs: Yes
F5-V-6 (8 M) *BBS2*: p.Arg315Gln	Maculopathy	W:36/H:128 BMI:22 (>95th percentile for age); rapid early-childhood gain; HP	None BL pes planus	Occipital flattening, hypertelorism, and broad nasal bridge (HC 55 cm)	Rt cryptorchidism Pre-puberty	Inattention, hyperactivity; ADHD features	H-XR: Normal F-XR: BL T4 and T5 Clino.	Renal normal The Lt testicle is observed in the Lt scrotal sac The Rt testicle is observed in the Rt lower inguinal canal	Major: RD, OB, and GU Minor: CF, MSK, and NB Beales: Yes ERNs: Yes
F6-IV-4 (29 F) *BBS2*: p.Arg315Gln	Advanced; likely CRD	W:41/H:163 BMI:15.4 early-onset obesity and HP in early childhood; now underweight	None	Micrognathia and tooth malocclusion (HC 51 cm)	Normal	Normal NB; Hyperhidrosis	H-XR: suspected Boutonniere deformities F-XR: Two Ph of the Rt T5; Small and short MP of the Lt T5; BL T5 Clino.	BL moderate HN was more prominent on the Rt	Major: RD, OB, and REN Minor: CF, and MSK Beales: Yes ERNs: Yes
F6-IV-5 (18 M) *BBS2*: p.Arg315Gln	Advanced; likely CRD	W:96/H:183 BMI:28.7 rapid; early-onset obesity; progressive into adolescence; HP	None Pes planus	Short nose, short neck, and micrognathia (HC 57 cm)	Normal	Mild speech delay and delayed walking; normal cognition; gross motor delay; delayed tooth eruptions	H-XR: Normal F-XR: BL two Ph of the T5	Normal Fullness of pelvicalyceal system BL due to a full urinary bladder	Major: RD and OB Minor: CF and MSK Beales: No ERNs: Yes
F7-VI-1 (34 F) *BBS5*: p.Arg56Gly	Significant maculopathy; likely CRD	W:65/H:156 BMI:26.7 Hx of early-onset obesity; stable in adulthood	BL UL&LL PAP (surgically removed) None	None (HC 52 cm)	Recurrent UTIs, hx of VUR; recurrent dysuria	Normal; mild social-skill impairment; inappropriate loud laughter	H-XR: Normal F-XR: Lt residual ossicles at the T5 resembling remnant of PAP and two Ph of the T5; Rt Clino on the T5; small bony exostosis observed at the head of the fifth metatarsal bone	Lt RC measuring 1.8 cm Rt RS largest 5 mm moderate BL HN Small size of the Lt kidney (7.7 cm) with decreased corticomedullary differentiation and a few focal cortical scars	Major: RD, OB, PAP, and REN Minor: NB and MSK Beales: Yes ERNs: Yes
F7-VI-2 (31 M) *BBS5*: p.Arg56Gly	Significant maculopathy; likely CRD	W:111/H:161 BMI:42.8 Rapid early-childhood gain; HP; progressive	BL UL&LL PAP (surgically removed) BL pes planus	Short neck (HC 56 cm)	Normal GU and puberty INR, hypothyroidism, and dyslipidemia	Normal NB; first word at the age of 3; HTN, snoring, and hyposmia	H-XR: Two Ph in the Rt F5 F-XR: Lt Clino of the T5; Rt residual ossicle at the T5 resembling the remnant of PAP	BL moderate HN Rt RC 3.2 × 2.6 cm	Major: RD, OB, PAP, and REN Minor: ENDO, CF, and MSK Beales: Yes ERNs: Yes
F8-II-4 (36 M) *CEP290:* p.Arg1465*and p.Glu2363Asp	Asymmetric disease Rt, plausible confounding factor. Lt, chorioretinal atrophy	W:107/H:182 BMI:32.3 overweight since infancy; progressive weight gain in adolescence; NAp	None Mild digital clubbing	High anterior hairline, epicanthal fold, and broad nasal bridge (HC 59 cm)	Normal	Mild social anxiety; social avoidance	H-XR: Normal F-XR: BL T5 Clino; BL short MP of the T5	Normal	Major: RD and OB Minor: CF, NB, and MSK Beales: No ERNs: No (VUS)
F9-IV-1 (26 F) *BBS1*: p.Arg160Gln	Maculopathy; likely CRD	At 26 years W:64/H:150 BMI:28.4; at 23 years (before bariatric sleeve surgery) W:92/H:150 BMI:40.9 Diet intervention; currently NAp	None Reports back disk issues and hand pain	Lt sided epicanthal fold (HC NA)	NA	NA NB IBS; emotional eating episode; bariatric sleeve surgery 2 years ago	NA	NA	Major: RD and OB Minor: CF Beales: No ERNs: Yes
F9-IV-5 (15 M) *BBS1*: p.Arg160Gln	Plausible CRD	W:80/H:160 BMI:31.3 obesity; progressive; currently NAp	None NA; Rt T5 inverted to the midline	None	Hx of cryptorchidism Pre-puberty	NA NB IBS	NA	NA	Major: RD, OB, and GU Minor: MSK Beales: No ERNs: Yes

1 Age at examination. Abbreviations: ADHD, attention-deficit/hyperactivity disorder; BBS, Bardet–Biedl Syndrome; BL, bilateral; BMI, body mass index; CF, craniofacial features; Clino, clinodactyly; CRD, cone–rod dystrophy; ENDO, endocrine and metabolic abnormalities (e.g., insulin resistance, diabetes mellitus, hypothyroidism, dyslipidemia, and polycystic ovarian syndrome); ERNs, European Reference Networks; ERP, extrarenal pelvis; F, female; F5, fifth finger; F-, foot; GDD, global developmental delay; GU, genital features; H, height; HC, head circumference; HN, hydronephrosis; HP, hyperphagia; HTN, hypertension; Hx, history; H-, hand; IBS, irritable bowel syndrome; ID, intellectual disability; INR, insulin resistance; IRD, inherited retinal disease; LL, lower limb; Lt, left; M, male; MP, middle phalanx; MSK, musculoskeletal features; NA, not available; NAp, normal appetite; NB, neurobehavioral features including behavioral, psychiatric, and associated neurologic features (e.g., social withdrawal, anxiety, depression, autism-like traits, and seizures); OB, obesity or abnormal weight trajectory; OSA, obstructive sleep apnea; PAP, post-axial polydactyly; PCOS, polycystic ovarian syndrome; Ph, phalanges; RC, renal cyst; RCD, rod–cone dystrophy; RD, retinal dystrophy; REN, renal diseases, including hydronephrosis and renal cysts as major features and neurogenic bladder as a minor feature; RS, renal stone(s); Rt, right; T2, second toe; T2DM, type 2 diabetes mellitus; T3, third toe; T4, fourth toe; T5, fifth toe; UL, upper limb; UT-US, urinary tract ultrasound; UTIs, urinary tract infections; VUR, vesicoureteral reflux; VUS, variant of uncertain significance; W, weight; XR, X-ray. Amino acids: Ala, alanine; Asp, aspartic acid; Glu, glutamic acid; Gln, glutamine; Arg, arginine; *, indicates a stop codon.

Eventually, using the Beales-derived, phenotype-first diagnostic framework, 11 patients from five families (F3–F7) were identified to have met the threshold for a clinical diagnosis of BBS (Table 2). These individuals carried variants in *CFAP418* (n = 2), *BBS2* (n = 7), and *BBS5* (n = 2). In contrast, when applying the molecular-first diagnostic classification of BBS recommended by the ERNs, 18 of 19 patients were observed to meet the diagnostic criteria of BBS (Table 2). The specific genotypic and phenotypic findings for the cohort are detailed below, organized by family groups sharing the same causative gene.

### 3.2 Families (F1–F3) sharing the same splice donor variant (c.155 + 1G>A) in *CFAP418*

After genetic testing of families F1–F3, we identified a homozygous pathogenic splice-donor variant (c.155 + 1G>A) in the *CFAP418* gene (Figure 1; Supplementary Figure S1).

**FIGURE 2:**
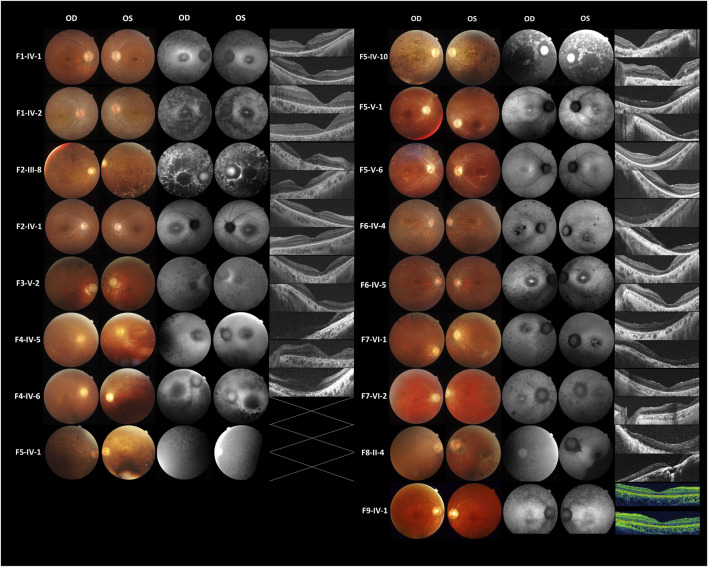
Multimodal retinal imaging in patients from families F1–F9. Each horizontal lane represents two patients, with the patients’ IDs located on the left and center of each lane. For each patient, images are shown for the right eye (OD) and left eye (OS), as labeled above the columns. Color fundus photographs are shown first, followed by fundus autofluorescence (FAF) images and corresponding macular optical coherence tomography (OCT) scans for the right eye (upper lane) and left eye (lower lane).

Family F1 presented with two affected siblings born to consanguineous first cousins (F1-IV-1, 17 years old (Y.O.); F1-IV-2, 15 Y.O.; Figure 1). Both first presented with nyctalopia, which later developed into hemeralopia, and total color blindness. Upon examination, their BCVA ranged from counting fingers at 0.5 m to 0.5, with greater interocular variability in F1-IV-2. Fundus examinations demonstrated the shared features of pale optic disc (POD), abnormal foveal reflection (FR) with pigment clumping (PC), attenuated blood vessels (ABVs), and peripheral PC in bony spicule-configuration, albeit more pronounced in the younger sibling (F1-IV-2). Concordantly, their FAF showed a central island of relatively preserved autofluorescence (AF) within the fovea, surrounded by a paracentral zone of hypo-AF and a perifoveal region of hyper-AF. Their OCT showed generalized macular thinning (MT), more pronounced in the periphery, with peripheral photoreceptor layer (PRL) loss and central speckled PRL (SPRL). Despite these shared features, the severity was greater in F1-IV-2. Noteworthy, ERG assessment of F1-IV-2 at age 10 (BCVA was 0.4 OU at that time) showed unrecordable signals from ffERG, while PERG assessment showed a reduction in P50 amplitude in both eyes, ranging from 71% (OD) to 85% (OS). The overall phenotype in family F1 was plausibly consistent with rod–cone dystrophy (RCD) (Figure 2).

**FIGURE 3:**
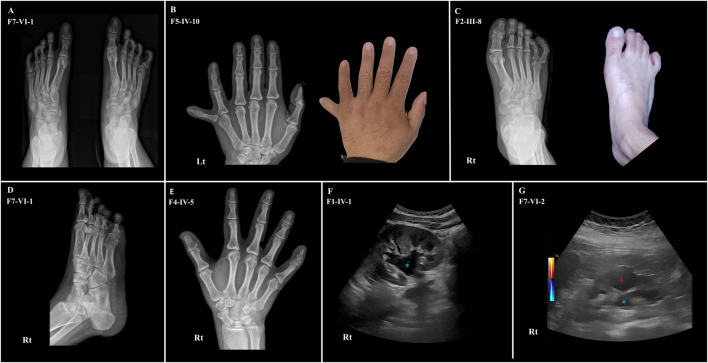
Representative images for hand x-ray, foot x-ray, selected clinical photographs of persistent polydactyly, and urinary tract ultrasound images. **(A)** Lt foot X-ray showing residual ossicle on the left fifth toe, resembling previous polydactyly, and a biphalangeal fifth toe. Rt foot X-ray showing clinodactyly of the right fifth toe. **(B)** Left hand x-ray and corresponding clinical photograph showing post-axial polydactyly of the Lt fifth finger. The sixth finger consisted of two phalanges. **(C)** Right foot x-ray and corresponding clinical photograph showing post-axial polydactyly of the Rt fifth toe, with the sixth toe consisting of two phalanges and clinodactyly of the fifth toe. **(D)** Bony small exostosis observed at the head of the fifth metatarsal bone, best appreciated on the oblique image. **(E)** Rt fifth finger clinodactyly, with a short, small middle phalanx. **(F)** Longitudinal Rt renal ultrasound image showing Rt moderate hydronephrosis (blue asterisk). **(G)** Longitudinal Rt renal ultrasound image showing moderate hydronephrosis (blue asterisk) and simple right renal cyst 3.2 × 2.6 cm (red asterisk). The corresponding patient IDs are shown in the upper left corner of each panel. Abbreviations: Rt, right; Lt, left.

F1-IV-2 had a BMI of 29.6, which is consistent with age-modified obesity, while F1-IV-1 maintained a normal weight life-long. F1-IV-2 presented with INR, dyslipidemia, and polycystic ovarian syndrome (PCOS). The mother reported prenatal renal cysts in F1-IV-1, without documented postnatal sequelae. Bilateral bi-phalangeal fifth toes along with bilateral fifth-toe clinodactyly were noted in F1 siblings. UT-US showed that F1-IV-1 exhibited moderate hydronephrosis (Figure 3), whereas F1-IV-2 had a left extrarenal pelvis with PCOS. Collectively, the siblings did not meet the diagnostic criteria for BBS according to the Beales-based framework and instead only reached the ERNs criteria, supporting a molecular-guided diagnosis of BBS.

The second family with the *CFAP418*:c.155 + 1G>A variant was F2. F2 was a two-generation family including two affected individuals: an uncle (F2-III-8; 36 Y.O.) and his niece, born to a third-degree consanguineous couple (F2-IV-1; 11 Y.O.) (Figure 1). Both initially experienced reduced vision and later developed hemeralopia, although the onset was earlier in the niece. Notably, nyctalopia presented a decade after the initial symptom onset in the uncle, while it remains absent in the niece to date. The latest BCVA ranged from no light perception (NLP) in the uncle to 0.2 in the niece.

The fundus examination in F2-III-8 demonstrated advanced IRD. Surprisingly, the niece’s (F2-IV-1) fundus appeared grossly normal in the posterior pole, with only subtle changes in the center of the fovea. In F2-IV-1, the far periphery of the fundus demonstrated subtle tessellated fundus (TF), ABV, and occasional PC. The FAF in F2-III-8 exhibited greater RPE loss than that of F2-IV-1, demonstrating confluent patches of hypo-AF throughout the macula. The niece’s FAF changes were confined to the macular area, showing a preserved central island of AF, surrounded by a hypo-AF rim and outer hyper-AF ring. Similarly, OCT scans showed severe MT with patchy loss of PRL in F2-III-8, while the niece had mild MT with only limited central PRL disruptions. The niece’s PERG responses were unrecordable, indicating macular dysfunction, and ffERG demonstrated amplitude losses ranging from 82.8% to 92.7% across all dark-adapted parameters, with unrecordable light-adapted responses. Collectively, the niece revealed evidence of cone–rod dystrophy (CRD), while her uncle showed an advanced IRD, likely within the CRD spectrum (Figure 2).

Both had a normal weight trajectory. The proband (F2-III-8) presented with persistent PAP (Figure 3), whereas the niece presented with micrognathia and unique bilateral triangular configuration of several distal toe phalanges, not observed elsewhere in this cohort. No additional major systemic features were identified. Similar to F1, the members of F2 met the diagnosis of BBS according to the ERNs framework, but not the Beales-based criteria (Table 2).

The last family with the *CFAP418*:c.155 + 1G>A variant was F3. Family F3 had several affected individuals with IRD across three generations (Figure 1). From the last affected generation, only one individual (F3-V-2; 44 Y.O.) agreed to undergo an ophthalmic examination (Figure 2). His early complaints included reduced vision, hemeralopia, and photophobia, which progressed to peripheral visual loss a decade later. He reported better night vision than daytime vision. F3-V-2 demonstrated an advanced IRD upon fundus examination. FAF imaging showed significant foveal RPE dysfunction, observed as confluent foveal hypo-AF areas, surrounded by patchy hypo-AF across the rest of the macula. Additionally, diffuse MT with irregular clumping of the PRL and a mild epiretinal membrane (ERM) were documented in his OCT. Together, F3-V-2 reflected an advanced IRD, with features suggesting CRD involvement.

The siblings (F3-V-2 and F3-V-4) from F3 exhibited abnormal weight trajectories. F3-V-2 had progressive obesity into adolescence, manifesting as truncal obesity. F3-V-4 had morbid obesity, with a BMI above 40 since childhood. Only F3-V-4 reported a history of PAP, unlike F3-V-2. In F3, family history indicated IRD with and without PAP among other relatives (Figure 1). F3-V-4 was also described as having hypothyroidism and type 2 diabetes mellitus. Both siblings also had dyslipidemia. F3-V-4 exhibited social withdrawal, depression, seizures, and abnormal choreiform movements. F3-V-2 showed milder psychiatric involvement, reporting chronic low mood managed with escitalopram. Left renal cyst, together with bilateral renal stones, was noted in F3-V-2 upon UT-US evaluation. Ultimately, the two siblings from F3 met the diagnosis of BBS based on both the Beales-driven and ERNs criteria (Table 2).

### 3.3 Families (F4–F6) harboring variants in *BBS2*

Upon molecular investigation, families F4–F6 were found to have two homozygous variants in *BBS2*. The first pathogenic variant (c.98C>A; p.Ala33Asp) in *BBS2* was identified in Family F4. F4 comprised two affected siblings (F4-IV-5, 32 Y.O.; F4-IV-6, 29 Y.O.; Figure 1) born to first-cousin parents. Both presented with hand motion (HM) visual acuity (VA), bilateral dense posterior subcapsular cataracts (PSCCs), nystagmus, and exotropia. The most recent fundus assessment in both showed a hazy view due to cataracts, with abnormal FR, POD, TF, ABV, and peripheral PC. FAF imaging in both revealed a comparable pattern of central macular hypo-AF surrounded by a rim of relatively increased AF. Their OCT scans demonstrated diffuse MT with an SPRL and mild ERM (Figure 2). Collectively, the findings in F4 pointed to a plausible significant maculopathy with features suggesting CRD involvement (Figure 2).

Both siblings of F4 displayed overlapping features, including early-onset obesity, PAP, pes cavus, gait abnormalities, genital hypoplasia, and vesicoureteral reflux (VUR). Moreover, they manifested with impaired neurodevelopment, ranging from impaired performance to social withdrawal, along with features suggestive of autism. F4-IV-5 had a history of cryptorchidism, along with hypertension, hypothyroidism, and focal seizure. F4-IV-6 had a history of unspecified renal disease and asthma. In F4, F-XR showed fifth-toe clinodactyly in both siblings. F4-IV-5 showed a residual ossicle at the left fifth toe, consistent with prior PAP. His hand X-ray (H-XR) also showed right fifth-finger clinodactyly and a small middle phalanx of the fifth right finger. However, F4-IV-6 revealed a normal H-XR. UT-US revealed left moderate hydronephrosis in F4-IV-6 with an absent ipsilateral jet, while F4-IV-5 exhibited bilateral extrarenal pelvis. Overall, all members from F4 met the diagnosis of BBS based on both Beales-based and ERNs frameworks (Table 2).

The other pathogenic variant in *BBS2* was identified in families F5 and F6 (c.944G>A; p.Arg315Gln). F5 comprised four affected individuals spanning two generations (Figure 1; Supplementary Figure S1). The older generation (F5-IV-10; 39 Y.O. and F5-IV-1; 53 Y.O.) had childhood-onset IRD, light perception VA, and ocular misalignment. Fundus examination in both showed POD, severe macular atrophy with irregular PC, diffuse TF, ABV, and PC involving the posterior pole and peripheral retina. Their FAF demonstrated confluent hypo-AF predominantly involving the macula. Overall, these findings indicated advanced IRD, likely favoring a CRD pattern (Figure 2).

The younger generation, who are cousins (F5-V-6; 8 Y.O and F5-V-1; 15 Y.O) had IRD onset before age 1. Upon ophthalmic examinations, both displayed reduced VA (0.2–0.4 VA) and exotropia. Fundus findings included POD, abnormal FR, and TF. ABV and peripheral PC were more evident in F5-V-1 than in F5-V-6. FAF in F5-V-1showed relative foveolar sparing with confluent mottled hypo-AF in the fovea, surrounded by scattered hypo-AF dots across the macula and midperiphery. In contrast, FAF in F5-V-6 revealed a subtle foveal hypo-AF area surrounded by a hyper-AF ring, with grossly preserved AF elsewhere. OCT in F5-V-1 demonstrated significant foveal thinning with generalized PRL disruption, more pronounced in the fovea than in the periphery. In contrast, OCT in F5-V-6 showed mild MT with mild ERM. The overall impression for both cousins (F5-V-6 and F5-V-1) favored maculopathy, with the older cousin (F5-V-1) showing more severe findings (Figure 2).

All recruited members in F5 had early-onset obesity, and INR was reported in three. PAP was observed only in F5-IV-10 (Figure 3). Craniofacial dysmorphism was present with variable expressivity in all family members. The niece demonstrated gait abnormalities and chronic joint pains, prompting rheumatologic evaluation. The genitourinary findings in F5 included right varicocele (F5-IV-10), right cryptorchidism (F5-V-6), and recurrent UT infection (UTI), with VUR and neurogenic bladder (F5-V-1). The neurodevelopmental and behavioral features were noted in all, except F5-IV-10. These ranged from mild behavioral symptoms (F5-V-6) to intellectual disability and impaired social functioning (F5-IV-1 and F5-V-1).

For family F5, H-XR revealed structural abnormalities in three individuals (F5-IV-1, F5-IV-10, and F5-V-1). A PAP of the left fifth finger, consisting of two phalanges, was documented in F5-IV-10 (Figure 3). F5-IV-1 showed brachymetacarpia of the right fourth finger and F5-V-1 had Boutonniere deformities. No visible H-XR anomalies were noted in F5-V-6. F-XR detected malformations in all individuals (F5-IV-10, F5-IV-1, F5-V1, and F5-V-6). F5-IV-10 and F5-V-6 had bilateral fifth-toe clinodactyly. F5-IV-10 also had a two-phalangeal left fifth toe with a short middle phalanx on the right fifth toe. F5-IV-1 had accessory bones in both feet. UT-US indicated features suggestive of chronic cystitis in two individuals (F5-IV-10 and F5-IV-1). Additionally, the right kidney of F5-IV-10 showed a renal cyst with a few angiomyolipomas, and his left kidney demonstrated a dromedary hump. UT-US in F5-V-1 revealed small bladder capacity and bladder wall thickening, consistent with neurogenic bladder. Together, based on the Beales-derived and ERNs criteria, the four members of F5 fulfilled the criteria for BBS diagnosis (Table 2).

Family F6 had the same causative variant observed in family F5 (*BBS2*:p.Arg315Gln). F6 presented with two affected siblings (F6-IV-4; 29 Y.O. and F6-IV-5; 18 Y.O.) from a third-degree consanguineous marriage (Figure 1). The older sibling (F6-IV-4) had HM VA, mild myopia, pendular nystagmus, and sensory exotropia. Her younger brother (F6-IV-5) had mild visual impairment (0.4 VA) with color blindness. Reported symptom onset differed, with reduced vision and hemeralopia at age 4 in the sister and at 10 in her brother.

Fundus findings included abnormally shaped POD, TF, foveal, and peripheral PC in both, yet more pronounced in F6-IV-4. RPE disturbance was noted on FAF in both, albeit with different patterns (Figure 2). F6-IV-4 showed patchy hypo-AF at the fovea, whereas F6-IV-5 showed a central hyper-AF area, surrounded by a hypo-AF ring, reflecting a severe macular involvement in both. The siblings’ OCT demonstrated generalized MT with diffuse PRL disruption. Collectively, the phenotype in family F6 was most consistent with CRD (Figure 2).

F6-IV-4 and F6-IV-5 exhibited different weight trajectories by adulthood. F6-IV-5 demonstrated early-onset obesity with progressive adolescent weight gain. In contrast, F6-IV-4 transitioned from early-childhood overweight to underweight in adulthood. Neurodevelopmentally, no major cognitive impairment was documented in either individual, although the brother had a history of early-onset gross motor and speech delays. Two-phalangeal fifth toes were noted in both, with different laterality. Only F6-IV-4 revealed a small middle phalanx of the fifth toe and bilateral fifth-toe clinodactyly. UT-US showed bilateral moderate hydronephrosis in F6-IV-4 and normal bladder fullness in F6-IV-5. Ultimately, F6-IV-4 met the BBS diagnostic criteria under both frameworks, whereas F6-IV-5 fulfilled the BBS diagnosis under ERNs metrics only (Table 2).

### 3.4 Family F7 with a variant in *BBS5*

The F7 pedigree comprised multi-generational consanguineous unions, with multiple affected individuals spanning four generations (Figure 1). To unravel the genetic basis of F7, we conducted molecular testing of F7’s siblings. A homozygous pathogenic variant in *BBS5* (c.166A>G; p.Arg56Gly) was identified. The two affected siblings (F7-VI-1, 34 Y.O and F7-VI-2, 31 Y.O) reported visual impairment since childhood. Each showed light perception to hand motion VA, and long-standing photosensitivity, with the younger brother noting relatively better peripheral than central vision. Their colored fundus demonstrated POD with peripapillary atrophy (PPA), absent FR with macular PC, TF, ABV, and mid-peripheral PC (Figure 2). FAF imaging revealed PPA and confluent foveal hypo-AF patches, with scattered hypo-AF spots observed throughout the remaining macula and midperiphery, suggesting marked maculopathy. OCT scans revealed diffuse MT with SPRL and mild ERM in both, yet more marked in F7-VI-1. Collectively, the F7 siblings’ phenotype suggested an advanced maculopathy within the CRD spectrum.

Both siblings had a history of early-onset obesity and PAP. Only F7-VI-1 complained of having recurrent UTIs, VUR, and dysuria. F7-VI-2 demonstrated INR, hypothyroidism, and dyslipidemia, along with hypertension. The neurodevelopmental outcomes ranged from normal to mild. H-XR findings were normal in F7-VI-1, while F7-VI-2 exhibited a two-phalangeal right fifth finger. Residual ossicles at the fifth toe, resembling a remnant of PAP and unilateral clinodactyly of the fifth toe, were observed in both individuals. F7-VI-1 also displayed a unique small bony exostosis at the base of the head of the fifth metatarsal bone. UT-US revealed that both displayed bilateral moderate hydronephrosis accompanied by renal cysts. Moreover, F7-VI-1 demonstrated a small left kidney (7.7 cm), decreased corticomedullary differentiation (CMD), and few cortical scars. Based on these findings, both siblings in F7 reached the BBS diagnostic thresholds according to the two BBS diagnostic frameworks (Table 2).

### 3.5 Family F8 with a variant in *CEP290*

Family F8 included a sporadic case (F8-II-4; 36 Y.O.), who underwent molecular testing to resolve the underlying IRD etiology in F8-II-4 (Figure 1; Supplementary Figure S1). Compound heterozygous variants were identified in *CEP290*, including a pathogenic nonsense variant (c.4393C>T; p.Arg1465*) and a variant of uncertain significance (c.7089A>T; p.Glu2363Asp). F8-II-4 demonstrated marked interocular asymmetry on ophthalmic examination. His BCVA ranged from HM in the right eye to 0.2 in the left eye.

Notably, early-onset strabismus, amblyopia, and PSCC were confined to the right eye. Fundus imaging revealed asymmetrical retinal findings. The right eye had hazy fundus views due to the presence of dense cataracts, with POD, ABV, TF, and confluent PC in the periphery, along with occasional small hypopigmented fleck-like spots. Corresponding FAF demonstrated confluent hypo-AF signals, and OCT exhibited diffuse MT with PRL disruption. These findings raised the possibility of prior confounding elements, including trauma or infection (Figure 2). In contrast, the left eye fundus showed PPA, mild ABV, occasional TF, and a well-demarcated temporal sectoral chorioretinal atrophic lesion extending into the fovea while sparing the remainder of the macula. FAF exhibited hypo-AF corresponding to the atrophic region, consistent with RPE loss, and OCT delineated a focal scar-like lesion corresponding to the chorioretinal atrophy, while the surrounding retina appeared structurally normal. Collectively, F8-II-4 presented with markedly asymmetric intraocular findings, likely influenced by confounding factors.

This patient had a lifelong history of weight gain beginning in infancy. He experienced progressive adolescent weight gain, reaching a BMI of 32.3 in adulthood. He exhibited no polydactyly, genitourinary, or endocrine features. His craniofacial findings included a high anterior hairline, epicanthal folds, and a broad nasal bridge. He showed mild digital clubbing during musculoskeletal examination, with no other structural abnormalities. He had preserved cognition but endorsed mild social avoidance and anxiety. F-XR in F8-II-4 showed bilateral fifth-toe clinodactyly, together with bilateral short middle phalanges in the same toe. Overall, F8-II-4 did not reach the BBS diagnostic thresholds for BBS under either the Beales-derived framework or the ERNs framework (Table 2).

### 3.6 Family F9 with a variant in *BBS1*

The third-degree consanguineous parents in Family F9 had two affected siblings (F9-IV-1; 26 Y.O and F9-IV-5; 15 Y.O. Figure 1; Supplementary Figure S1). Genetic analysis revealed a homozygous pathogenic variant (c.479G>A; p.Arg160Gln) in *BBS1*. Family F9 declined formal clinical evaluation; therefore, only self-reported information and prior ophthalmic records were available. Both siblings first presented with childhood-onset reduced VA. Fundus imaging of F9-IV-1 at age 17 showed an apparently normal optic disc, early macular pigmentary changes, and subtle TF without ABV. Concurrently, her FAF demonstrated mild PPA, confluent foveal hypo-AF with an outer hyper-AF rim, and patchy AF across the macula and midperiphery (Figure 2). OCT scans showed generalized MT with SPRL. Furthermore, her ERG showed extinguished scotopic and photopic responses, along with an unrecordable PERG. These findings in F9-IV-1 were consistent with maculopathy, evolving to CRD. Regarding their weight trajectory, F9-IV-1 had childhood-onset weight gain, with peak obesity reaching a BMI of 40.9 at 23 years, which improved after bariatric sleeve surgery. F9-IV-5 had normal early childhood weight, followed by progressive weight gain, resulting in obesity (BMI 31.3). He had a history of corrected childhood cryptorchidism. No other major clinical manifestations were reported in either individual. According to the ERNs recommendations, both F9 siblings satisfy the BBS diagnosis criteria; however, neither of them met the Beales-based metrics (Table 2).

## 4 Discussion

We investigated the molecular etiology of 19 patients from nine families initially diagnosed with presumed non-syndromic IRD. Interestingly, genetic testing revealed variants in genes known to be associated with either isolated IRD or BBS. The discovered genes included *CFAP418* (n = 3 families), *BBS2* (n = 3), *BBS5* (n = 1), *BBS1* (n = 1), and *CEP290* (n = 1). These findings prompted a reverse-phenotyping assessment strategy, through which affected individuals were systemically reassessed for extra-ocular manifestations. This reverse phenotyping revealed previously unrecognized features in several individuals that could be part of a broader ciliopathy spectrum. We observed intra- and inter-familial variability in the presenting features, even among patients harboring the same gene or identical variants. To our knowledge, reverse phenotyping for BBS has not previously been reported in Jordanian IRD cohorts to assess for overlooked syndromic involvement. Furthermore, given the rarity of reported BBS cases with *CFAP418* as the underlying etiology, our findings contribute to expanding the clinical spectrum of this gene. Hence, we present the most comprehensive extra-ocular assessment of patients carrying *CFAP41*8 so far.

Within our cohort, the most recurrent causative variant was found in *CFAP418,* identified in three unrelated Jordanian families (F1–F3). *CFAP418* has been previously associated with isolated IRD, including RCD, CRD, and maculopathy, and has been more recently implicated in BBS (Estrada-Cuzcano et al., 2012; van Huet et al., 2013; Goyal et al., 2023; Clark et al., 2024). Functionally, *CFAP418* has been shown to regulate membrane lipid homeostasis, a process essential for maintaining mitochondrial morphology and vesicular trafficking within cilia (Yang et al., 2022; Clark et al., 2024). Disruption of membrane lipid homeostasis has been proposed to cause IRDs and ciliopathies (Clark et al., 2024).

*CFAP418* has been rarely reported in patients with BBS (Caba et al., 2022). Thus far, *CFAP418* has been implicated in only two confirmed cases of BBS, with two additional cases reported as being suspected BBS (Estrada-Cuzcano et al., 2012; Heon et al., 2016; Khan et al., 2016). The scarcity of BBS cases with *CFAP418* reflects either true rarity or potential underdiagnosis, necessitating further characterization of patients with presumed non-syndromic IRD carrying *CFAP418* variants. In alignment, the ERNs emphasized the importance of reporting additional BBS cases involving *CFAP418* (Dollfus et al., 2024).

We sought to perform reverse extra-ocular phenotypic characterizations for three families (F1–F3) carrying *CFAP418*, initially presumed to have non-syndromic IRD. Notably, two of the families described herein (F2 and F3) were previously reported as part of the Jordanian IRD cohort, yet their extra-retinal features were not investigated (Azab et al., 2021). So far, the *CFAP418:*c.155 + 1G>A variant identified in families F2 and F3 has not been reported outside the prior Jordanian IRD cohort.

Patients from families F1–F3 harboring the *CFAP418:*c.155 + 1G>A variant showed variable retinal and extra-retinal phenotypes. Specifically, a spectrum of IRD was observed, ranging from RCD in F1 to CRD in F2 and F3. In F1, even among close-aged siblings, the phenotype was more severe in the younger sibling than in her older brother. A similarly broad IRD spectrum has been described in earlier studies (Estrada-Cuzcano et al., 2012; van Huet et al., 2013; Goyal et al., 2023; Clark et al., 2024). Together, these findings highlight marked intra- and inter-familial variability in *CFAP418*-associated disease and indicate that *CFAP418*-related retinal involvement is not confined to a single, well-defined pattern. Nevertheless, here, two of the three families harboring this *CFAP418* variant demonstrated phenotypes skewed toward a CRD-like presentation. To this extent, the previously reported patients with BBS carrying *CFAP418* had likewise shown a tendency toward CRD (Estrada-Cuzcano et al., 2012; Heon et al., 2016; Khan et al., 2016). Therefore, we suggest that *CFAP418* may predispose a CRD-like phenotype within a broader IRD spectrum.

Driven by our genotype-guided secondary evaluation of extraocular features, diverse BBS-related manifestations were identified in families carrying *CFAP418*. Obesity, a primary feature of BBS, was observed in three patients from two families (F1 and F3). Even within the same family, F1, obesity was noted in the younger sibling, unlike her older brother. These observations emphasize both inter- and intra-familial variability in weight trajectory among patients with *CFAP418*. Similarly, the two previously confirmed *CFAP418*-induced BBS cases had abnormal weight trajectories (Heon et al., 2016; Khan et al., 2016). In contrast, one report described two adolescent siblings with CRD and polydactyly harboring *CFAP418* variants without describing their weight status (Estrada-Cuzcano et al., 2012). This absence of reported weight data may reflect either a normal weight trajectory or incomplete phenotypic characterization. Given the limited knowledge of the extraocular features in IRD patients with *CFAP418*, there is no clear association between the weight trajectory and this gene. This warrants an expanded characterization of the extraocular features in patients with *CFAP418* to reveal potential multisystemic features, as recommended by ERNs (Dollfus et al., 2024).

Polydactyly is another primary sign of BBS, observed in two of three *CFAP418*-associated families (F2 and F3), with intrafamilial variability in its presence. Particularly, the uncle in F2 and the older sister in F3 presented with polydactyly, unlike their other relatives included in the study. Interestingly, the previously described cases with putative *CFAP418*-related BBS uniformly exhibited polydactyly, albeit with variable laterality and anatomical distribution, as noticed in our cases (Estrada-Cuzcano et al., 2012; Heon et al., 2016; Khan et al., 2016). Additional BBS-associated manifestations previously described in the literature for patients with *CFAP418* included speech delay, mild learning difficulties, abnormal dentation, and elevated liver enzyme levels (Heon et al., 2016; Khan et al., 2016). However, none of the patients in our cohort exhibited these neurodevelopmental or dental features, and because they lacked hepatic symptoms, screening for liver enzymes was not performed. In contrast, the neuropsychiatric features were observed only in one of the three *CFAP418*-related families (F3), described as having depression, seizures, and social withdrawal, among others. The differences further highlight the marked clinical heterogeneity associated with *CFAP418*.

Given the potential under-recognition of renal and skeletal phenotypes, UT-US and H-F-XR were performed in our cohort. The *CFAP418*-associated renal involvements ranged from normal findings to renal cysts, extrarenal pelvis, and hydronephrosis, showcasing intra- and inter-familial variability. Notably, a published case report described that *CFAP418* was incidentally found to have a horseshoe kidney, despite normal renal function (Heon et al., 2016). These findings support the need for lifelong renal surveillance in patients carrying *CFAP418*-related variants, as recommended by ERNs (Dollfus et al., 2024). H-XR demonstrated consistently unremarkable results for all tested patients with *CFAP418*. Nevertheless, F-XR varied extensively, extending from PAP to more subtle abnormalities of the fifth toe. To our knowledge, our investigation documented the most comprehensive BBS-related phenotyping for patients with *CFAP418* as the underlying molecular culprit (Estrada-Cuzcano et al., 2012; Heon et al., 2016; Khan et al., 2016).

According to the classical clinical-guided classification of BBS, four major features or three major and two minor features are required to establish the diagnosis of BBS (Forsyth and Gunay-Aygun, 2023). Only the siblings in F3 met the classical BBS diagnostic criteria among the *CFAP418*-related families. However, the reliance on the classical diagnosis of BBS is hampered by the age-dependent expression of features and the wide variable expressivity among affected individuals (Dollfus et al., 2024). In contrast, the updated 2024 recommendations from ERNs emphasized the use of a genotype-first approach for diagnosing BBS. To fulfill the diagnosis by the proposed recommendations, positive molecular testing, along with the presence of one or two primary features, depending on the patient’s age, is required (Dollfus et al., 2024). Consequently, all patients from families F1–F3 met the diagnosis of BBS. These findings underscore the limitations of the traditional, phenotype-only approach, which can obscure the proper diagnosis of patients with BBS and their management.

The remaining families harbored variants in *BBS1*, *BBS2*, *BBS5,* and *CEP290*. The assembly of three proteins encoded by the genes *BBS1*, *BBS2,* and *BBS5*, along with five other proteins, forms an octamer structure. This structure is called the BBSome complex, which is essential for the regulation of ciliary signaling and transportation (Tian et al., 2023). Pathogenic variants in genes responsible for the formation of the BBSome complex can lead to the manifestation of either an IRD-only phenotype or BBS.

Interestingly, three families (F4–F6) consisting of eight patients harbored causative variants in *BBS2*. These cases had an ophthalmic impression that was most consistent with either maculopathy or CRD. Prior cases with the same identified variants in *BBS2* were described in the context of retinitis pigmentosa and CRD (Katsanis et al., 2001; Beales et al., 2003; Eichers et al., 2004; Shevach et al., 2015; Kousi et al., 2020; Sharon et al., 2020; Marinakis et al., 2021; Meng et al., 2021; Milibari et al., 2024; Schmidt et al., 2024; Zacchia et al., 2025). All eight patients with *BBS2* also experienced a history of obesity. Three patients from two families (F4 and F5) had a history of polydactyly. The intellectual disability was documented in four out of eight patients. Our findings further underscore the variable expressivity of *BBS2*-related features. According to the phenotype-guided classification of BBS, seven of eight patients with *BBS2* met the threshold for BBS diagnosis (Forsyth and Gunay- Aygun, 2023). Prior studies reported the variants described herein in *BBS2* in either isolated IRD or BBS (Katsanis et al., 2001; Beales et al., 2003; Eichers et al., 2004; Zaghloul et al., 2010; Shevach et al., 2015; Kousi et al., 2020; Sharon et al., 2020; Marinakis et al., 2021; Meng et al., 2021; Milibari et al., 2024; Schmidt et al., 2024; Zacchia et al., 2025). After revising the diagnosis by adopting the recent recommendations from ERNs, all patients met the diagnostic criteria for BBS (Dollfus et al., 2024). In concordance, a prior meta-analysis of patients harboring *BBS2* reported a high syndromic score, along with demonstrating high polydactyly penetrance compared to other genes (Niederlova et al., 2019). Notably, earlier reports indicate that patients with the same variant in F4 (*BBS2*:p.Ala33Asp) have been described to have both non-syndromic IRD and BBS (Shevach et al., 2015; Sharon et al., 2020; Marinakis et al., 2021; Schmidt et al., 2024). However, none of the cases with the variant *BBS2*:p.Ala33Asp were described in sufficient detail to meet the traditional BBS diagnostic criteria (Shevach et al., 2015; Sharon et al., 2020; Marinakis et al., 2021; Schmidt et al., 2024). Here, we performed comprehensive phenotyping for patients with variants in *BBS2*, which can help in augmenting evidence for future genotype–phenotype association studies.

Beyond *CFAP418* and *BBS2*, the remaining families in our work harbored variants in *BBS1*, *BBS5*, and *CEP290*, each identified in F9, F7, and F8, respectively. Both siblings from F7, who had *BBS5* as the causative variant, met the two diagnostic frameworks for BBS diagnosis (Forsyth and Gunay-Aygun, 2023; Dollfus et al., 2024). Despite prior reporting of the *BBS5*:p.Arg56Gly variant, detailed phenotyping has not been described (Muller et al., 2010; Mary et al., 2019; Kousi et al., 2020). Patients with variants in *BBS5* have been described to show a propensity for syndromic presentation (Niederlova et al., 2019). The remaining patients from families F8 and F9, harboring variants in *CEP290* and *BBS1*, respectively, did not reach the recommended threshold for classical diagnosis of BBS. The same *BBS1*:p.Arg160Gln variant identified in F9 has been previously described in both syndromic and non-syndromic presentations, emphasizing the clinical heterogeneity associated with *BBS1* (Schmid et al., 2011; Breuel et al., 2019; Isabelle et al., 2020; Orlova et al., 2024; Ozguc Caliskan et al., 2024). Notably, variants in *BBS1* have been proposed to have milder phenotypes compared to other BBS-related genes (Daniels et al., 2012; Niederlova et al., 2019). According to the molecular-guided diagnosis of BBS, both siblings in F9 can be diagnosed with BBS (Dollfus et al., 2024). The sporadic case in F8 carrying candidate biallelic variants in *CEP290* did not satisfy the molecular-guided diagnosis of BBS as one variant was classified as pathogenic, while the second remained of uncertain significance (Dollfus et al., 2024).

We acknowledge several limitations of this study. The retrospective design resulted in non-uniform diagnostic evaluations and heterogeneous clinical data across patients, which might have affected the consistency and interpretation of genotype–phenotype correlations. Additionally, some assessments relied on self-reported data or were performed at different institutions using varying methodologies. Future prospective studies employing standardized, multidisciplinary protocols are warranted to more accurately delineate the phenotypic spectrum of BBS-related genes.

In this study, we recruited nine families comprising 19 patients with an initial diagnosis of non-syndromic IRD. Molecular testing revealed variants in genes previously implicated in both isolated IRD and BBS. Consequently, to uncover potentially overlooked syndromic features, we adopted a genotype-guided reverse phenotyping approach. This strategy identified previously unrecognized BBS-related features and revealed marked intra- and inter-familial phenotypic variability. Consequently, 11 patients from five families were reclassified as having BBS under traditional diagnostic criteria. Notably, our findings illustrated the limitations of the traditional phenotype-first diagnosis framework of BBS. By adopting the molecular-driven diagnostic approach recommended by ERNs, 18 of 19 patients reached the diagnostic threshold for BBS. Collectively, these results demonstrated that the BBS-related features underlie a continuum of clinical manifestations rather than a dichotomous division between syndromic and isolated IRD. To our knowledge, this represents the first molecular investigation of BBS in Jordan. To date, our work provides the most comprehensive phenotypic characterization of *CFAP418*-associated disease within the BBS spectrum. Our results support adopting genotype-driven evaluation as the foundation for diagnosis and management of BBS-related diseases.

## Data Availability

The original contributions presented in the study are included in the article/Supplementary Material; further inquiries can be directed to the corresponding authors.

## Glossary

**ABV**: attenuated blood vasculature
**ACMG**: American College of Medical Genetics and Genomics
**ADHD**: attention-deficit/hyperactivity disorder
**AF**: autofluorescence
**AM**: AlphaMissense score
**BBS**: Bardet–Biedl syndrome
**BCVA**: best-corrected visual acuity
**BL**: bilateral
**BMI**: body mass index
**CF**: craniofacial features
**Clino**: clinodactyly
**CMD**: corticomedullary differentiation
**ComHET**: compound heterozygous
**Co-seg.**: co-segregation analysis
**CRD**: cone–rod dystrophy
**EEG**: Electroencephalography
**ENDO**: endocrine and metabolic abnormalities
**ERG**: electroretinography
**ERM**: epiretinal membrane
**ERNs**: InterEuropean Reference Networks
**ERP**: extrarenal pelvis
**F**: female
**F-**: foot
**F5**: fifth finger
**FAF**: fundus autofluorescence imaging
**ffERG**: full-field electroretinography
**FR**: foveal reflection
**GDD**: global developmental delay
**gnomAD**: Genome Aggregation Database
**GU**: genital features
**H**: height
**HC**: head circumference
**hg19**: human genome assembly GRCh37
**hg38**: human genome assembly GRCh38
**HGVS**: Human Genome Variation Society
**HM**: hand motion
**HN**: hydronephrosis
**HOM**: homozygous
**HP**: hyperphagia
**HTN**: hypertension
**Hx**: history
**H-**: hand
**IBS**: irritable bowel syndrome
**ID**: intellectual disability
**INR**: insulin resistance
**IRB**: Institutional Review Boards
**IRDs**: inherited retinal diseases
**ISCEV**: International Society for Clinical Electrophysiology of Vision
**LL**: lower limb
**LP**: likely pathogenic
**Lt**: left
**M**: male
**MAF**: minor allele frequency
**MP**: middle phalanx
**MSK**: musculoskeletal features
**MT**: macular thinning
**Mut**: mutant
**n**: numbers
**NA**: not available
**NAp**: normal appetite
**NB**: neurobehavioral features
**NLP**: no light perception
**OB**: obesity or abnormal weight trajectory
**OCT**: optical coherence tomography
**OD**: right eye (oculus dexter)
**OS**: left eye (oculus sinister)
**OSA**: obstructive sleep apnea
**P**: pathogenic
**PAP**: post-axial polydactyly
**PC**: pigment clumping
**PCOS**: polycystic ovary syndrome
**PERG**: pattern electroretinography
**Ph**: phalanges
**POD**: pale optic disc
**PPA**: peripapillary atrophy
**PRL**: photoreceptor layer
**PSCC**: posterior subcapsular cataracts
**RL**: REVEL score
**RC**: renal cyst
**RCD**: rod–cone dystrophy
**RD**: retinal dystrophy
**REN**: renal disease
**RPE**: retinal pigment epithelium
**RS**: renal stone(s)
**Rt**: right
**SPRL**: speckled photoreceptor layer
**T2DM**: type 2 diabetes mellitus
**T3**: third toe
**T4**: fourth toe
**T5**: fifth toe
**TF**: tessellated fundus
**UL**: upper limb
**US**: ultrasound
**UT**: urinary tract
**UTIs**: urinary tract infections
**v2**: gnomAD version 2.1.1
**v4**: gnomAD version v4.1.1
**VA**: visual acuity
**VUR**: vesicoureteral reflux
**VUS**: variant of uncertain significance
**W**: weight
**WES**: whole-exome sequencing
**WT**: wild-type
**XR**: X-ray
**Y.O.**: years old.
Amino acids
A (Ala): alanine
**R (Arg)**: arginine
**D (Asp)**: aspartic acid
**Q (Gln)**: glutamine
**E (Glu)**: glutamic acid
**G (Gly)**: glycine
*****: indicates a stop codon
