# Supplementary material for: Clinical heterogeneity associated with Bardet–Biedl syndrome-related genes in presumed non-syndromic inherited retinal disease
Source: Front Cell Dev Biol. 2026 Jun 2;14:1802945. doi: 10.3389/fcell.2026.1802945 (PMC13269086; doi:10.3389/fcell.2026.1802945)
Supplement: Supplementary file 1 [file DataSheet1.pdf]

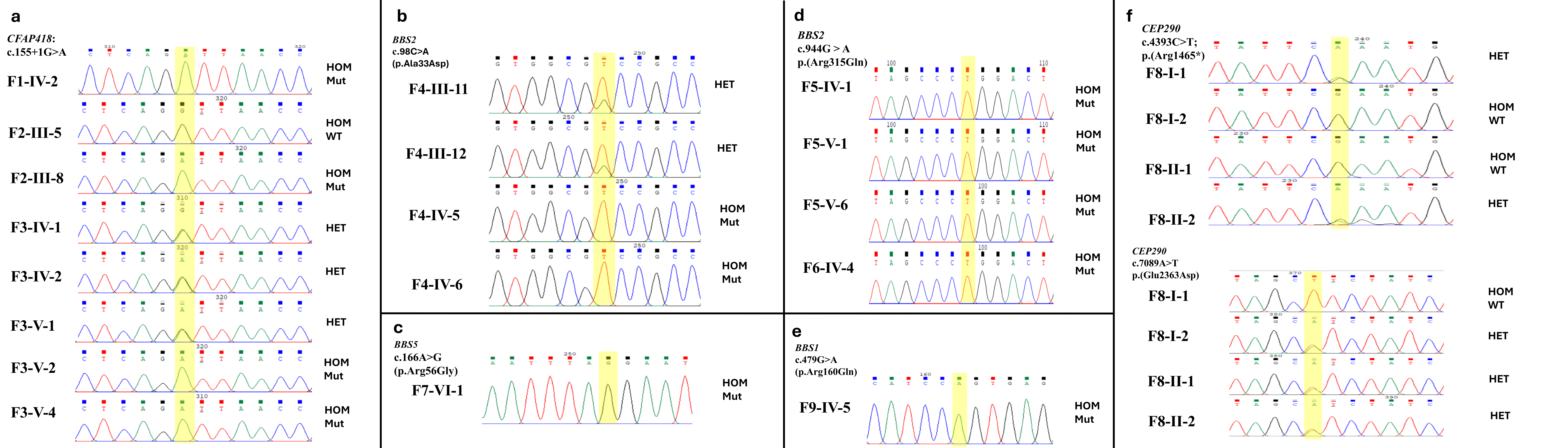
a
CFAP418: c.155+1G>A
F1-IV-2
HOM Mut
F2-III-5
HOM WT
F2-III-8
HOM Mut
F3-IV-1
HET
F3-IV-2
HET
F3-V-1
HET
F3-V-2
HOM Mut
F3-V-4
HOM Mut
b
BBS2 c.98C>A (p.Ala33Asp)
F4-III-11
HET
F4-III-12
HET
F4-IV-5
HOM Mut
F4-IV-6
HOM Mut
c
BBS5 c.166A>G (p.Arg56Gly)
F7-VI-1
HOM Mut
d
BBS2 c.944G > A p.(Arg315Gln)
F5-IV-1
HOM Mut
F5-V-1
HOM Mut
F5-V-6
HOM Mut
F6-IV-4
HOM Mut
e
BBS1 c.479G>A (p.Arg160Gln)
F9-IV-5
HOM Mut
f
CEP290 c.4393C>T; p.(Arg1465*)
F8-I-1
HET
F8-I-2
HOM WT
F8-II-1
HOM WT
F8-II-2
HET
CEP290 c.7089A>T p.(Glu2363Asp)
F8-I-1
HOM WT
F8-I-2
HET
F8-II-1
HET
F8-II-2
HET

Supplementary Figure S1. Sanger sequencing chromatograms validating the identified variants and demonstrating their co-segregation with disease status in the proband, affected relatives, and unaffected family members from families F1–F9 harboring variants in Bardet–Biedl syndrome-related genes. (a) chromatogram illustrating NM_177965.4 (*CFAP418*):c.155+1G>A variant in families F1, F2 and F3. (b) chromatogram showing NM_031885.5 (*BBS2*):c.98C>A; p.Ala33Asp variant in family F4. (c) chromatogram illustrating NM_152384.3(*BBS5*):c.166A>G (p.Arg56Gly) variant in family F7. (d) chromatogram illustrating NM_031885.5 (*BBS2*):c.944G>A (p.Arg315Gln) variant in families F5 and F6. (e) chromatogram of illustrating NM_024649.5 (*BBS1*):c.479G>A; p.Arg160Gln variant in family F9. (f) chromatogram illustrating NM_025114.3 (*CEP290*):c.4393C > T; p.Arg1465 * and NM_025114.3(*CEP290*):c.7089A > T; p.Glu2363Asp variant in family F8. HET, heterozygous; HOM, homozygous; Mut, mutant; WT, wild type
